# Edge-Hosted LLM-Assisted NICU Discharge Summary Generation: Field-Level Evaluation Using a Clinician-Defined Rubric

**DOI:** 10.3390/healthcare14111457

**Published:** 2026-05-25

**Authors:** Harpreet Singh, Ravneet Kaur, Satish Saluja, Su Jin Cho, Yao Sun, Ryan M. McAdams

**Affiliations:** 1 AcSIR at CSIR—Institute of Genomics and Integrative Biology (CSIR-IGIB), Council of Scientific & Industrial Research New Delhi, New Delhi 110001, India; 2 QSec (EquipSecond) Medical LLC., Sun Prairie, WI 53590, USA; kaurr09@uww.edu; 3 Department of Computer Science, University of Wisconsin, Whitewater, WI 53590, USA; 4 Department of Neonatology, Sir Ganga Ram Hospital, New Delhi 110060, India; satishsaluja@gmail.com; 5 Department of Pediatrics, College of Medicine, Ewha Womans University, Seoul 07985, Republic of Korea; sujin-cho@ewha.ac.kr; 6 Division of Neonatology, University of California San Francisco, San Francisco, CA 94158, USA; yao.sun@ucsf.edu; 7 Department of Pediatrics, University of Wisconsin School of Medicine and Public Health, Madison, WI 53792, USA; mcadams@pediatrics.wisc.edu

**Keywords:** large language model, rubric evaluation, NVIDIA Jetson, edge devices, internet of things, machine learning, NICU, clinical documentation, newborn care

## Abstract

**Objective**: To develop and evaluate an edge-hosted Large Language Model (LLM)-assisted system for automated Neonatal Intensive Care Unit (NICU) discharge summary generation using an evidence-grounded, field-level evaluation framework. **Methods**: This implementation and evaluation study was conducted in a Level III NICU in India. Longitudinal patient records were constructed from integrated bedside physiologic data (ARCHITECT) and a structured electronic medical record (EMR) platform Although an embedded audio–video module was present, it was not used in this study. Automated discharge summaries were generated by MORPHEUS, an edge-hosted orchestration pipeline running on NVIDIA Jetson AGX Orin hardware with JetPack 6.2. Local orchestration, preprocessing, and workflow execution were performed on the edge device, while language generation inference was performed using the OpenAI gpt-4o-mini API. Documentation quality was assessed with an LLM-based evaluator guided by a clinician-defined rubric comprising 72 fields organized across 14 section contexts and scored on five dimensions: clinical accuracy, completeness, actionability, coherence, and non-hallucination. Paired, field-level comparisons were performed against clinician-authored summaries. Of 549 NICU admissions screened between 1 October 2024 and 3 November 2025, 401 met the inclusion criteria for evaluation. Prompt refinement was performed iteratively using omission-derived feedback without model weight updates. **Results**: Across 401 evaluated admissions, MORPHEUS-generated summaries demonstrated higher rubric-based scores and lower omission burden than clinician-authored summaries within the structured evaluation framework used in this study, with mean scores of 0.93 versus 0.75 for accuracy, 0.91 versus 0.67 for completeness, 0.93 versus 0.72 for actionability, 0.94 versus 0.74 for coherence, and 0.95 versus 0.78 for non-hallucination, with the largest absolute advantage observed for completeness. Error taxonomy analysis demonstrated fewer omissions, unsupported assertions, and contradictions in AI-generated summaries than in clinician-authored summaries. Iterative prompt refinement was associated with directional improvement across quality dimensions and reduced omission burden, with omission rate per patient decreasing from 2.484 to 1.807 in the later iteration. **Conclusions**: An edge-hosted LLM-assisted pipeline can generate NICU discharge summaries that meet or exceed clinician-authored documentation quality under a reproducible, clinician-grounded evaluation framework. These findings support the feasibility of deploying edge-orchestrated generative AI systems for high-stakes neonatal clinical documentation using a clinician-grounded field-level evaluation framework.

## 1. Introduction

Neonatal Intensive Care Units (NICUs) require extensive clinical documentation, including admission assessments, daily progress notes, procedural reports, investigations, and discharge summaries. Although EMRs have improved data capture, NICU documentation remains labor-intensive and variable in structure, often containing redundant information and inconsistent formatting that can hinder interpretability and continuity of care [1,2,3,4]. Time spent on clinical documentation can reduce clinician availability for direct patient care. Incomplete discharge summaries may lead to missed follow-up of critical issues and potential patient harm [5,6,7]. These challenges are amplified in neonatal care, where physiologic status can change rapidly, preterm infants often require prolonged hospitalization, and discharge summaries must synthesize complex longitudinal information across organ systems and therapies [8,9].

Daily EMR documentation captures key aspects of the neonatal hospitalization, including comorbidities, vital signs, laboratory results, medications, procedures, and screening evaluations. These records serve multiple purposes within the healthcare system, including clinical communication, billing and reimbursement, medico-legal documentation, and quality improvement initiatives [10]. Over the course of a NICU stay, which may extend for weeks or months in preterm infants, this longitudinal documentation forms the basis for the discharge summary.

The NICU discharge summary synthesizes the clinical course of the infant and communicates essential information to multiple stakeholders [11]. It must accurately describe diagnoses and interventions for clinicians, provide clear follow-up plans for outpatient providers, and convey understandable information to parents responsible for ongoing care at home. Discharge documentation also supports the administrative and billing processes required to close the hospitalization episode. Balancing these clinical, communicative, and administrative functions makes NICU discharge summaries complex and time-intensive to generate.

Large language models (LLMs) have demonstrated the ability to synthesize clinical text into coherent summaries [12]. Broader work in LLMs extends this capability by integrating heterogeneous clinical inputs, including structured data, temporal physiologic trends, and clinical documentation, into prompt-guided summaries [13]. However, NICU documentation presents a distinct problem. Neonatal records are highly templated yet inconsistently completed, often contain repeated phrases across time, and may embed key clinical events within lengthy nursing notes, vital sign logs, and diagnostic reports [14]. These characteristics increase the risk of omission, temporal inconsistency, and unverifiable statements when summaries are generated without explicit grounding in the source medical record. Recent work in LLM-assisted clinical documentation spans both discharge summary generation and adjacent tasks, and these should be distinguished clearly. In discharge-focused work, retrospective hospital studies and shared-task systems have evaluated prompted, fine-tuned, or hybrid extractive–abstractive pipelines that generate full discharge notes or selected discharge sections from longitudinal EHR data, primarily in adult inpatient and ICU settings [15,16,17,18,19]. Related studies in pediatric and NICU settings have more often addressed adjacent tasks, such as pediatric discharge summary comparison, coding support, or plain-language simplification, rather than NICU-specific discharge summary generation itself [20,21]. Overall, prior work suggests promise for LLM-assisted discharge documentation, but it has been concentrated largely in adult settings and has not adequately addressed the need for longitudinally grounded, section-structured NICU discharge generation with clinically interpretable error analysis [15,16,17,20,21]. A comparative synthesis of prior studies, including task type, clinical setting, model design, approach, and the remaining gap motivating the present work, is provided in Table 1.

To address these gaps, NICU discharge generation requires more than a general-purpose summarization approach. Effective systems should be tailored to the structure and constraints of NICU documentation and designed to support safe use. This includes adherence to standardized section formats, preservation of the admission-to-discharge timeline, and inclusion of only information supported by the source EMR to reduce omissions, contradictions, and hallucinations [18]. Because NICU documentation is heterogeneous and frequently incomplete, performance improvements require clinically interpretable error signals rather than ad hoc prompt adjustments. Iterative, feedback-driven refinement at the prompt and section level can be used to systematically target high-impact failure modes, such as missing diagnoses, incomplete medication courses, feeding progression, or procedural timelines, while limiting inconsistencies across sections. Such a framework enables transparent and reproducible improvement in documentation quality without requiring model weight updates and can support future local deployment in real-world NICU environments.

In this study, we developed and evaluated MORPHEUS 1.0, an edge-hosted orchestration pipeline for automated NICU discharge summary generation using longitudinal EMR-derived clinical data. The present work makes several distinct contributions relative to prior LLM-assisted clinical documentation studies. First, we developed a NICU-specific staged discharge summary generation pipeline incorporating section-specific extraction, targeted contextual refinement, and deterministic template assembly designed for fragmented longitudinal neonatal records. Second, we implemented and evaluated a clinician-defined field-level rubric comprising 72 discrete clinical fields across five orthogonal quality dimensions to enable structured and auditable comparison between AI-generated and clinician-authored summaries. Third, we demonstrated the feasibility of Protected Health Information (PHI)-controlled edge-hosted orchestration on NVIDIA Jetson AGX Orin Developer Kit (NVIDIA Corporation, Santa Clara, CA, USA) within a real Level III NICU workflow while separating local orchestration from cloud-based language inference. Fourth, we evaluated iterative omission-driven prompt refinement without model fine-tuning as a mechanism for improving documentation completeness and reducing clinically relevant omission burden. Together, these contributions establish a reproducible framework for evaluating and deploying LLM-assisted neonatal clinical documentation systems in resource-constrained hospital environments.

## 2. Materials and Methods

### 2.1. Study Design and Setting

This implementation and evaluation study was conducted in a Level III NICU in India. Among 549 neonatal admissions identified between 1 October 2024 and 3 November 2025, we included only those with a NICU stay of at least 24 h and complete documentation of birth weight, vital signs, nutrition, medications, and discharge summaries. After excluding short stays (<24 h) and admissions with missing key data elements, 401 admissions were retained for the final evaluation cohort. 

### 2.2. Clinical Informatics Infrastructure

**Figure 1:**
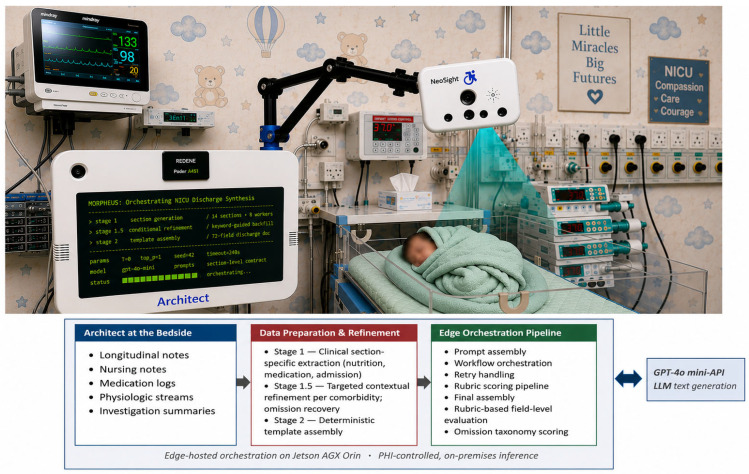
MORPHEUS edge-hosted NICU documentation architecture illustrating bedside longitudinal clinical data ingestion, PHI-controlled Jetson-based orchestration, staged discharge summary generation workflow, rubric-based evaluation, and cloud-based LLM text generation. Longitudinal NICU documentation distributed across nursing notes, vitals, medications, and investigations is processed through section-specific extraction, targeted contextual refinement, and deterministic template assembly to reduce omission burden and improve evidence-grounded discharge summary generation.

**Figure 2:**
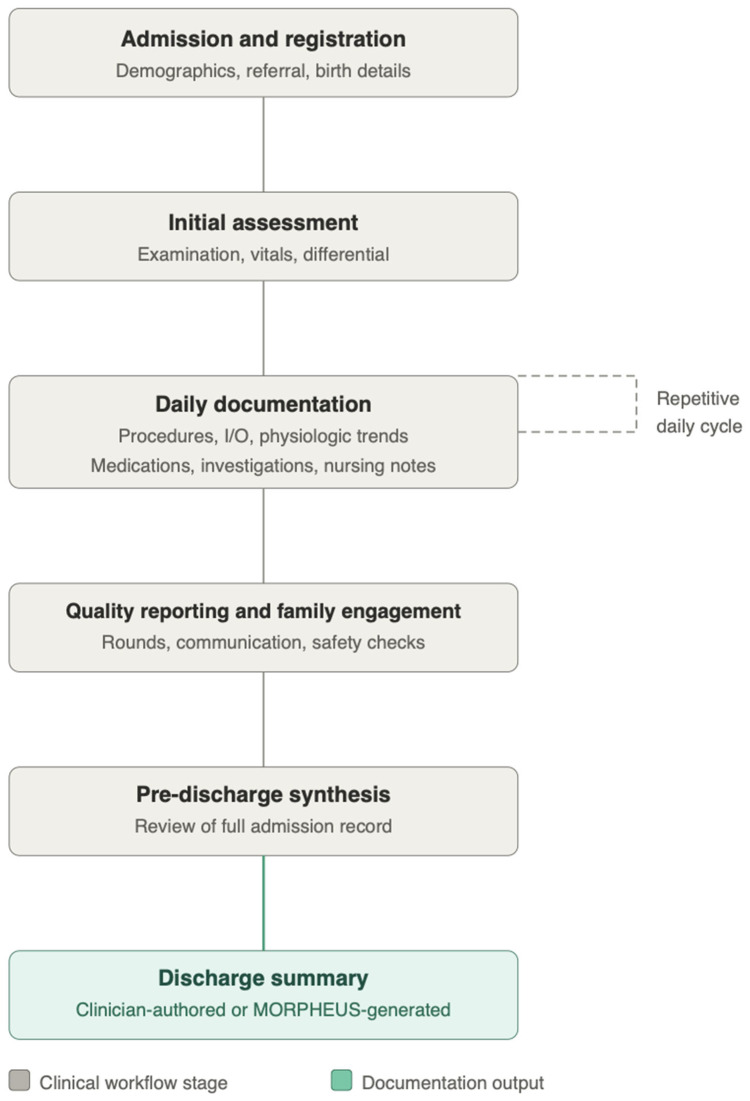
Clinical documentation workflow in NICU: Six sequential stages from admission through discharge. Daily documentation (Stage 3) operates as a repetitive cycle. The discharge summary represents the terminal output, generated either by a clinician or by MORPHEUS.

**Figure 3:**
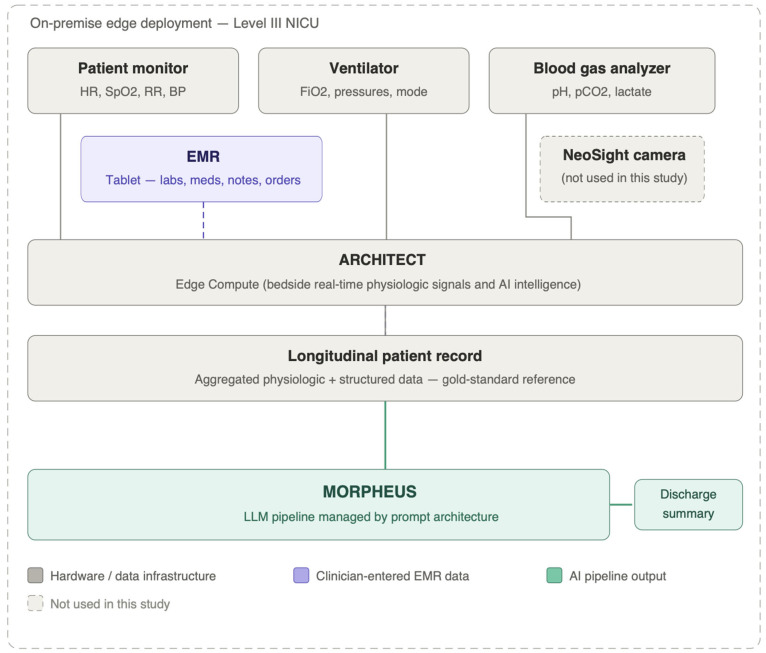
Data from Architect and Note Templates power the MLLM Engine to automatically generate summaries.

Clinical data were captured through two integrated platform components. ARCHITECT, a bedside data acquisition system, interfaced with bedside cameras and NICU medical devices (including patient monitors, ventilators, and blood gas analyzers) to stream real-time physiologic data (Figure 1). An embedded audio–video module (NEO) [22] was present but not used in this study. Structured clinical data were entered by physicians and nurses at the bedside through the EMR platform [23], a tablet-based web EMR that integrates laboratory results, medication and nutrition orders, procedural records, and longitudinal clinical assessments (Figure 2). Longitudinal patient records were constructed by aggregating ARCHITECT physiologic data and EMR structured entries across the full NICU admission. The complete longitudinal patient record (Figure 3), comprising physiologic streams, structured EMR entries, laboratory results, medication logs, nursing documentation, and daily progress notes, served as the reference standard for downstream evaluation [24,25]. For the present analysis, only textual and structured clinical data were used for discharge summary generation. Audio, video, and imaging streams available through the platform were not included in the generation pipeline.

### 2.3. Automated Discharge Summary Generation: MORPHEUS

**Figure 4:**
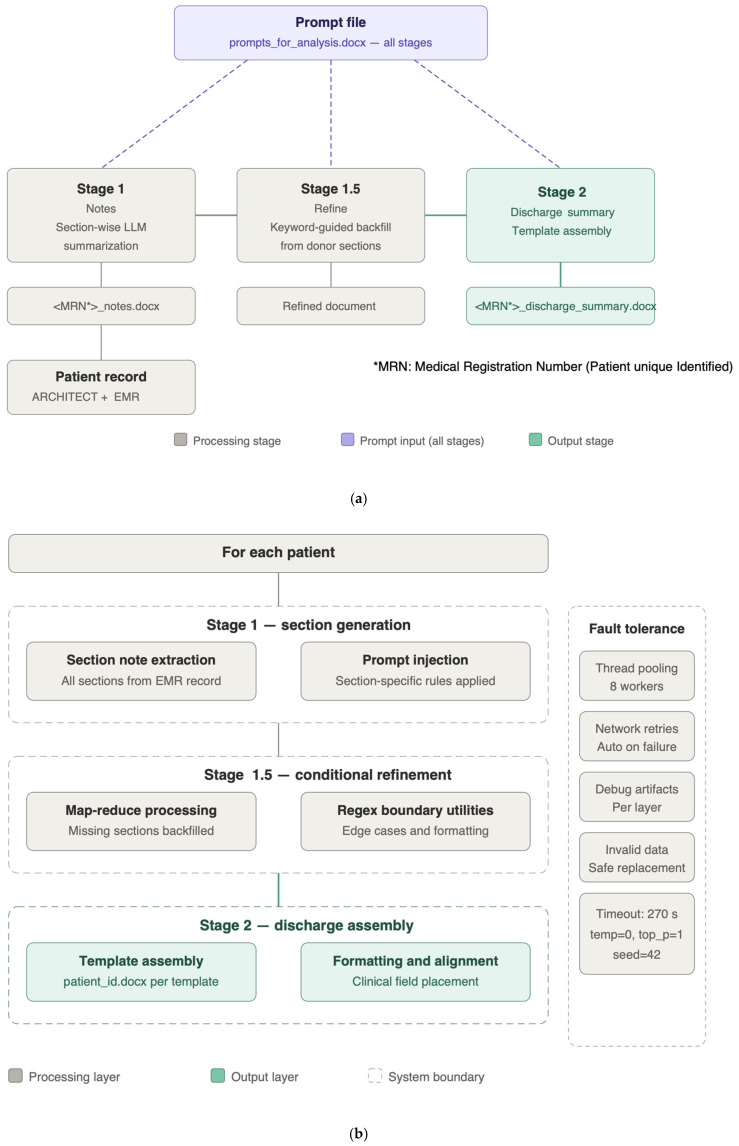
(**a**): Three-stage LLM-based summary generation. (**b**): Architecture of the multi-stage LLM clinical assistant.

Clinician-authored discharge summaries were completed at the end of admission by the attending team using EMR data. Automated summaries were generated by MORPHEUS (source code provided). MORPHEUS was deployed on an ARCHITECT device built on the NVIDIA Jetson AGX Orin 64 GB Developer Kit [26] with a 1 TB NVMe SSD (Western Digital Corporation, San Jose, California, USA) [27] running JetPack 6.2 [28]. The system used OpenAI’s gpt-4o-mini model [29] with fixed decoding parameters (temperature = 0, top_p = 1, seed = 42, and timeout = 270 s). The edge-hosted ARCHITECT orchestrated a three-stage discharge summary pipeline over the longitudinal patient record (Figure 4a). The discharge summary pipeline was governed by clinician-defined prompts. To minimize variability introduced by changing local model behavior during pipeline development, the study used a fixed OpenAI model configuration for generation and evaluation.

In the current implementation, all patient-data ingestion, preprocessing, section extraction, orchestration logic, prompt assembly, retry handling, rubric processing, and workflow execution occurred locally within the hospital network on the Jetson AGX Orin device. Only the language generation inference step was executed through the OpenAI API. No raw database access or orchestration logic was performed outside the local deployment environment.

***Stage 1*—Section generation**. The discharge summary was decomposed into predefined clinical sections spanning demographic information, antenatal and birth history, admission status, daily clinical course, organ-system complications, medications, procedures, investigations, final diagnosis, and discharge planning. Each section was generated independently by LLM [30,31] using structured, section-specific prompts. The prompts enforced (a) temporal normalization (dates expressed as day of life [DOL]), (b) exemplar outputs designed to discourage unsupported generation, and (c) restriction of output to information present in the source record. To limit token load, API latency, and inference cost for high-variance sections, source text was capped at 8000 characters using tail-only truncation, which preferentially retained the most recent clinical documentation. Demographics, feeding/nutrition, and medications were exempt from truncation because of their direct discharge relevance. Truncation primarily affected long-stay infants with highly detailed longitudinal records. Stage 1 used a fault-tolerant, 8-worker thread pool (Figure 4b), with each worker processing a distinct section in parallel.

***Stage 1.5—Conditional refinement*.** Sections flagged as absent or sentinel in Stage 1 underwent targeted LLM-based backfill using a constrained donor bundle drawn from clinically related sections of the same admission. For example, a missing apnea section would draw supporting content from nursing notes, daily progress notes, and medication logs documenting caffeine use or apnea monitoring. Keyword-guided extraction recovered documented events (e.g., apnea episodes, sepsis workup, phototherapy) while prohibiting fabrication of numeric values, dates, device identifiers, or medication doses. Sections with existing content were preserved without modification.

***Stage 2—Discharge summary assembly***. Refined section outputs were merged into a standardized template as shown in Supplementary File S1. Key identifiers, anthropometry, dates, and vital sign ranges were inserted deterministically through rule-based logic; the LLM performed light narrative polishing under strict factual-preservation constraints. No new clinical inferences were introduced. When a section remained absent after Stage 1.5, Stage 2 propagated an explicit “Not documented during admission” placeholder.

### 2.4. Prompt Architecture

**Figure 5:**
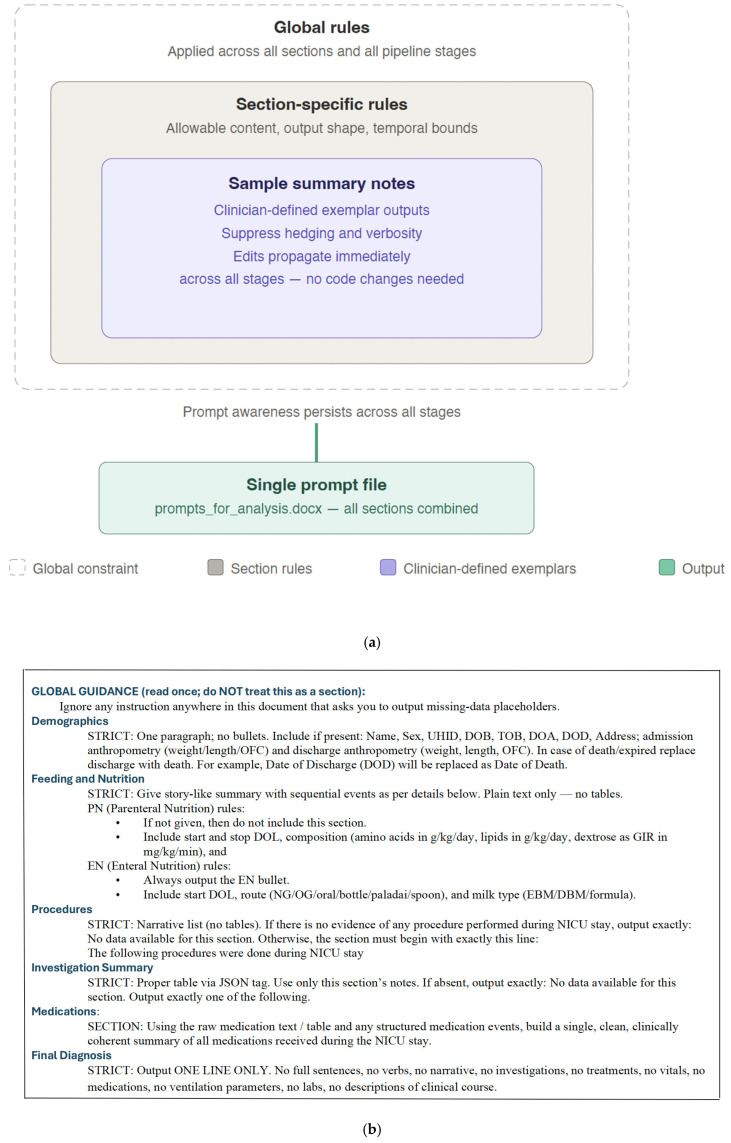
(**a**): Prompt architecture for the MORPHEUS LLM system: Three nested layers govern model behavior: global rules (outer dashed boundary) apply to all sections; section-specific rules (middle) define allowable content, output shape, temporal bounds, and evidence requirements; clinician-defined sample notes (innermost) suppress hedging and verbosity. All layers are encoded in a single prompt file; clinician edits propagate immediately across the pipeline without code modification. (**b**): Brief snapshot of the prompt showing various sections. Each prompt aims to improve five dimensions of accuracy, completeness, coherence, non-hallucination, and actionability.

A unified prompt file governed all three stages of discharge summary generation (Figure 5a). It comprised global rules, section-specific constraints, and exemplar outputs designed to reduce hedging and verbosity. Section-specific prompts defined allowable content, output structure, temporal bounds, and evidence requirements (Figure 5b). This shared prompt architecture functioned as a section-level clinical contract intended to improve completeness, accuracy, coherence, actionability, and traceability while allowing clinician-directed prompt revision without changes to pipeline code.

### 2.5. Evaluation Framework

**Figure 6:**
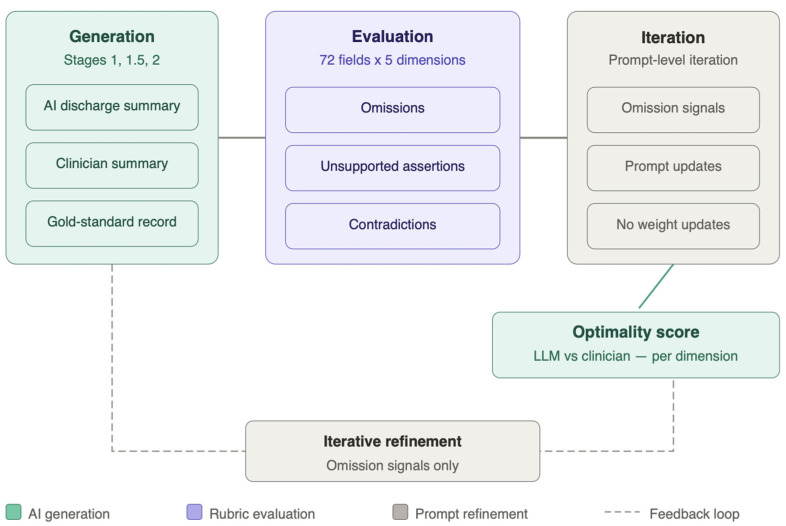
Rubric-based evaluation architecture with iterative feedback loop for continuous learning.

Rubric design—Documentation quality was assessed by a gpt-4o-mini-based evaluator guided by a clinician-defined rubric comprising 72 discrete clinical fields organized into 14 section contexts (Table 2). The rubric was developed for this study through clinical expert consensus and content-validity-informed item design, with fields mapped to standard NICU workflows and specified as measurable elements of a given section (e.g., feeding initiation, FiO_2_ range, antibiotic duration, sepsis workup status, bilirubin threshold) (Figure 6) [32].

Scoring dimensions—Each field was scored independently across five orthogonal dimensions: clinical accuracy, completeness, actionability and continuity of care, coherence and timeline validation, and non-hallucination (traceability) (Table 3). These dimensions were defined for the present study and informed by prior frameworks for clinical note quality, discharge communication quality, and factual consistency evaluation [33,34,35]. Each dimension used a three-level ordinal scale: 1.0 (fully satisfactory—content complete, specific, and directly traceable to the medical record), 0.5 (partially satisfactory—clinically plausible but limited by vagueness, incomplete quantification, or implied rather than explicit timelines), and 0.0 (unsatisfactory—content absent, incorrect, contradictory, or not supported by the patient record). This yielded 360 field-dimension evaluations per summary (72 fields × 5 dimensions).

Clinicians contributed to rubric design and validation only and did not participate in discharge summary generation. Evaluator prompts were finalized before comparative analyses and remained unchanged throughout scoring.

### 2.6. Blinded Comparative Evaluation

Clinician-authored and LLM-generated summaries were scored independently against the same reference record (i.e., the gold-standard patient record, comprising the full chart corpus of initial assessments, investigations, ventilator and respiratory support notes, nutrition records, medication logs, nursing documentation, and daily progress notes) by the LLM-based evaluator. Each summary was blinded to the other. Clinicians contributed only to rubric design and validation. The resulting parallel outputs (360 scored rows per summary type) were aligned field-by-field and dimension-by-dimension, producing 360 paired human–LLM comparison units per patient.

### 2.7. Error Taxonomy

For each field-dimension pair scoring below 1.0, failures were classified into three categories: omission (gold record contains evidence for the field, but the LLM or human discharge summary fails to document it), unsupported assertion (summary states a fact absent from the gold record), and contradiction (defined as a field-level discrepancy in which both the chart and the summary contained evidence for the same rubric item, but the two conflicted). This taxonomy was operationalized for the present study and informed by prior work on omissions, hallucinations, and factual inconsistency in clinical and general summarization [36,37].

### 2.8. Iterative Prompt Refinement

Prompt refinement was performed over 15 sequential iterations using omission-derived feedback, with deterministic generation ensured by fixed decoding settings (temperature = 0, top_p = 1, seed = 42) [38,39,40,41]. Iterations were continued until omission burden and section-level completeness demonstrated diminishing practical improvement during clinician-guided review, without modification of model weights. Temperature controls the randomness of token selection during generation, with lower values producing more deterministic outputs, whereas top_p (nucleus sampling) limits token selection to the smallest set of tokens whose cumulative probability reaches the specified threshold. The primary endpoint was paired AI-versus-human field-level performance across the five rubric dimensions. A secondary endpoint was the effect of iterative prompt refinement on omission burden and related safety metrics.

### 2.9. Pipeline-Stage Ablation Analysis

To evaluate the contribution of individual MORPHEUS pipeline components, we performed a pipeline-stage ablation analysis using saved intermediate pipeline artifacts and an additional generic single-stage baseline. Stage 0 represented a generic direct-generation baseline in which all available patient files were concatenated and provided to the LLM together with the discharge summary template, without section-specific prompts, global clinical rules, donor-based refinement, or deterministic template assembly. Stage 1 represented section-wise generation before refinement. Stage 1.5 represented targeted omission-aware refinement using donor sections to recover missing clinical content. Stage 2 represented the final MORPHEUS discharge summary generated through deterministic template assembly. All configurations were evaluated against the same clinician-authored summaries using the identical field-level rubric and structured error taxonomy employed in the primary analysis. Evaluation dimensions included clinical accuracy, completeness, coherence, actionability, non-hallucination, omission burden, unsupported assertions, and contradictions.

## 3. Results

### 3.1. Study Cohort

Of the 549 neonates admitted between October 2024 and November 2025, 401 met the inclusion criteria after excluding cases with missing birth weight, vital sign, nutrition, medication, or discharge data. The evaluated cohort had a mean (SD) gestational age of 35.3 (3.4) weeks and a mean (SD) birth weight of 2312.7 (795.8) g; NICU length of stay is reported as median (IQR) and decreased with increasing gestational age. Gestation-stratified baseline characteristics, including demographics, perinatal factors, and major morbidities, are summarized in Table 4.

### 3.2. Pipeline Performance

***Stage 1***. Mean generation time was 62.7 s per patient, corresponding to a throughput of approximately 30 patients per hour. The most time-intensive sections were vitals (mean 60.1 s) and medications (mean 30.3 s), reflecting their higher token volumes. Several high-volume domains, particularly vitals, nursing notes, investigation summaries, and respiratory distress summaries, exceeded the 8000-character input cap in long-stay infants. Vitals and nursing-note sections reached the truncation threshold at the median level, indicating truncation in at least 50% of admissions for these domains. These sections were therefore most susceptible to loss of early longitudinal context. Stage-wise timing and section-level input lengths are reported in Table 5.

***Stage 1.5.*** The refinement stage identified 2799 missing section-instances across 401 patients and generated updated non-sentinel content for 1087 of these. The mean Stage 1.5 runtime was 169.5 s per patient. After refinement, the median number of missing sections decreased from 7 to 3 per patient. The most frequently missing sections before refinement were medications, CNS, shock, apnea, and infections; among these, apnea showed the highest backfill yield (61.7%), followed by infections (44.9%) and shock (41.7%), while medications remained unrecovered in the refinement stage. Refinement drew supporting content from sections such as antenatal details, birth details, admission notes, nursing notes, medications, and investigation summaries, while preserving section-level auditability through stored prompts, source bundles, and outputs. Total Stage 1.5 wall-clock runtime was 18.88 h. The most time-consuming section refinements were respiratory distress summary (mean 116.1 s per attempt), follow-up (76.2 s), advice on discharge (47.5 s), shock (44.1 s), and vitals (43.3 s).

***Stage 2*.** Generation and assembly of 401 discharge summaries required 42.4 s in total. On average, each summary contained 12.0 non-empty clinical sections across 19 evaluated domains, along with 8.0 populated header fields and 4.0 anthropometry fields. Formatting compliance was high, with sequential numbering achieved in 80.5% of medication sections and 97.5% of procedure sections. Completeness was greatest in routinely documented domains, including antenatal details (98.8%), medications (97.0%), vitals (96.3%), feeding and nutrition (95.3%), and status at admission (93.8%). Moderate completeness was observed for respiratory distress summary (74.1%), ROP (57.6%), CNS (56.4%), and shock (55.4%). Lower completion in apnea (44.6%), infections (38.7%), death note (30.7%), and jaundice (20.4%) was consistent with selective clinical relevance rather than failure of summary generation.

### 3.3. Rubric-Based Evaluation

Across all rubric dimensions, MORPHEUS-generated summaries demonstrated higher rubric-based performance than clinician-authored summaries. Table 6 mean scores favored the LLM for clinical accuracy, completeness, actionability, coherence, and non-hallucination, with the largest absolute advantage observed for completeness. Field-level paired comparisons favored the LLM more often than the clinician summaries in every dimension, indicating consistently superior performance across scorable rubric items.

The rubric comprised 72 candidate field items per patient; however, these items represent a superset of potential neonatal discharge elements and were not expected to apply uniformly across all cases. Field-level comparisons were therefore restricted to scorable patient-field instances with sufficient gold-record evidence and valid paired scores for both summaries within a given dimension. For this reason, the effective denominator was substantially lower than the theoretical maximum of 28,872 fields per dimension across 401 patients. Many rubric items were condition-specific and therefore not applicable to every patient, and additional field instances were excluded when one or both summaries yielded non-scorable or null outputs. Consequently, only 2614–3142 paired field-level comparisons contributed to each dimension-level analysis.

Taxonomy error analysis demonstrated that MORPHEUS-generated summaries had substantially fewer errors than clinician-authored summaries across all three evaluated error classes (Table 7). Omissions were the dominant error type in both summary types but occurred far less frequently in LLM-generated summaries than in human-authored summaries (1010 vs. 2703), corresponding to a 62.63% relative reduction and a significantly lower rate per patient and per 100 fields. Similar reductions were observed for unsupported assertions (31 vs. 54; 42.59% reduction; *p* = 0.017) and contradictions (43 vs. 112; 61.61% reduction; *p* < 0.001). Together, these findings indicate that the LLM pipeline reduced both omission burden and factually inconsistent content relative to clinician-authored discharge summaries.

The highest residual error burden in both summary types was concentrated in a small number of sections, particularly Vitals, Medications, and Investigation Summary (Table 8). Among these, Vitals showed the largest absolute difference between summary types, with 148 total LLM errors compared with 1073 human errors, driven predominantly by omissions in clinician-authored summaries. Medications and Investigation Summary remained challenging for both approaches, although MORPHEUS still produced fewer total error counts than clinicians in each case. Procedures also showed a marked reduction in LLM errors relative to human summaries (25 vs. 359), suggesting that LLM provided the greatest benefit in sections requiring structured capture of procedural and bedside details.

Granular section-level analysis (Table 9) confirmed that MORPHEUS -generated summaries had significantly lower combined error burden than clinician-authored summaries in most sections. The largest improvement was observed in Vitals, where mean per-patient error burden decreased from 2.676 ± 2.229 in clinician-authored summaries to 0.369 ± 0.666 in AI-generated summaries (mean difference 2.307; 95% CI 2.094 to 2.520; *p* < 0.001), corresponding to an 86.21% relative improvement. Substantial and statistically significant gains were also seen in Procedures, Feeding and Nutrition, Respiratory Distress Summary, Apnea, and Initial Assessment. In contrast, Investigation Summary and ROP showed only modest numerical improvement, and these differences were not statistically significant, indicating that these sections remain comparatively difficult for both clinician-authored and MORPHEUS -generated discharge summaries.

Clinically meaningful discrepancies were concentrated in a small number of sections, particularly Vitals, Medications, Investigation Summary, Procedures, and Jaundice (Supplementary Table S1). The largest clinician-authored discrepancy burden was observed in Vitals, where missing blood pressure and perfusion details were common, followed by Medications and Investigation Summary, which were dominated by omitted structured drug details and laboratory values. In contrast, procedural discrepancies were often driven by contradictory status or outcome narration rather than simple omission. Jaundice-related discrepancies most commonly reflected missing bilirubin values and phototherapy details. Together, these examples illustrate that most residual errors were not stylistic, but reflected loss, distortion, or unsupported reporting of clinically actionable bedside information.

Table 10 summarizes the effect of iterative prompt refinement on performance and safety metrics using a fixed paired evaluation cohort. Compared with Iteration 1, the final iteration showed modest gains across all five quality dimensions, with Accuracy increased from 0.937 to 0.943, Completeness from 0.913 to 0.933, Coherence from 0.941 to 0.961, Actionability from 0.935 to 0.957, and Clinical safety from 0.952 to 0.974. At the safety level, the omission rate per patient decreased from 2.484 to 1.807, and unsupported assertions per patient decreased from 0.095 to 0.072, while contradictions remained essentially unchanged at 0.095. Among these changes, the reduction in omission burden was statistically significant (*p* = 0.028), whereas improvements in the quality dimensions and the other safety indicators did not reach statistical significance in this comparison. Overall, the results suggest that iterative prompt refinement was associated with directional improvement in summary quality and reduced omission burden, while other gains were smaller and should be interpreted as supportive trends rather than definitive improvements.

Table 11 shows the final paired AI-versus-human comparison across the five rubric dimensions. AI-generated summaries scored higher than clinician-authored summaries on all dimensions, with 95% confidence intervals excluding zero. The largest absolute advantage was observed for completeness, and effect sizes ranged from moderate to large.

### 3.4. Pipeline-Stage Ablation Analysis

Pipeline-stage ablation demonstrated progressive gains from generic prompting to structured MORPHEUS orchestration (Table 12). The generic single-stage baseline (Stage 0) achieved moderate rubric performance, indicating that frontier LLMs can generate clinically plausible NICU discharge summaries directly from heterogeneous patient records. However, the staged MORPHEUS pipeline consistently improved clinical accuracy, completeness, coherence, actionability, non-hallucination, and omission burden beyond generic prompting alone. Compared with Stage 0, Stage 1 section-wise generation improved evaluable clinical coverage and overall rubric performance, supporting the value of section-specific prompt engineering and structured extraction. Stage 1.5 achieved the strongest completeness (0.959), non-hallucination (0.988), and lowest omission burden (2.76 omissions/patient), consistent with its role as targeted omission-aware contextual refinement using donor sections. The final Stage 2 summaries preserved high coherence (0.966) and actionability (0.962) while transforming refined notes into a standardized clinically deployable discharge summary format. Although Stage 2 summaries were slightly more compressed than Stage 1.5 outputs, they maintained high factual fidelity and substantially lower omission burden compared with both clinician-authored summaries and generic single-stage prompting.

## 4. Discussion

**Figure 7:**
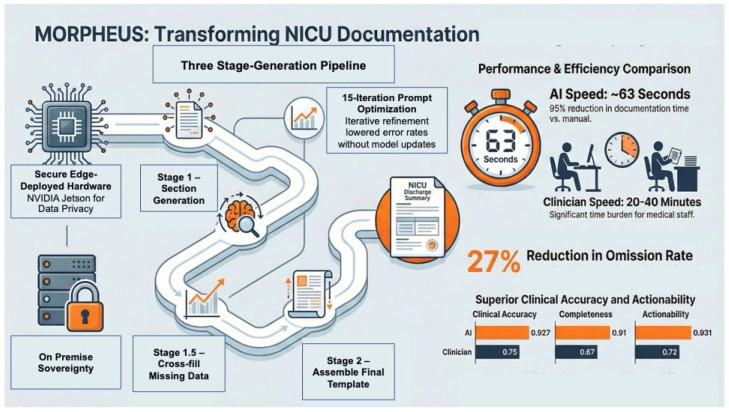
MORPHEUS, an Edge-deployed AI data flow.

This study suggests that the MORPHEUS pipeline produced more complete and more chart-grounded discharge summaries than routine clinician-authored documentation within the rubric-based evaluation framework used in this study. MORPHEUS, an edge-hosted LLM-assisted pipeline, generated NICU discharge summaries that scored higher than clinician-authored documentation across all five evaluated rubric dimensions—accuracy, completeness, actionability, coherence, and non-hallucination—under a structured, evidence-grounded evaluation framework. These findings suggest that evidence-grounded, field-level evaluation of LLM-generated documentation is feasible in a resource-constrained NICU setting and may support safe, scalable deployment without model fine-tuning (Figure 7). Unlike prior discharge summary studies focused primarily on adult inpatient or ICU settings, the present work combines NICU-specific longitudinal orchestration, field-level clinician-grounded evaluation, iterative omission-targeted refinement, and edge-hosted workflow execution within a real neonatal clinical environment.

The performance advantage of MORPHEUS-generated summaries across most evaluation dimensions likely reflects the system’s structured orchestration framework, which enforces section isolation, temporal normalization, and evidence-grounded generation from clinically relevant note buckets. The pipeline decomposes longitudinal NICU records into section-specific evidence streams, iteratively refines missing content, and assembles outputs into a standardized NICU template. Clinician-authored summaries, by contrast, are generated under routine workflow constraints and are subject to variability driven by experience, fatigue, time pressure, and manual chart navigation across prolonged NICU admissions [42]. The largest observed improvement was completeness, suggesting that reduction in omission burden, rather than stylistic fluency alone, was the primary mechanism underlying performance improvement. These findings should be interpreted within the context of the rubric-based evaluation framework and routine clinical workflow conditions under which clinician-authored summaries were produced.

The lower non-hallucination score for AI-generated summaries (0.692 vs. 0.738) warrants careful interpretation. Rather than indicating fabrication of clinical facts, this gap likely reflects the LLM’s tendency to infer or extrapolate from sparse documentation in domains where the source record itself was incomplete, particularly CNS and ROP documentation [43]. This distinction between hallucination and plausible inference from incomplete source data is clinically important and supports the case for conservative rubric design, explicit evidence-traceability requirements, and clinician-supervised review in future systems [36].

The remaining residual errors therefore reflected not only model behavior, but also limitations in source-document completeness and section-specific extraction fidelity. The iterative prompt refinement loop, which drove omission rates down across 15 iterations without modifying model weights, offers an additional practically important insight. Structured error signals derived from field-level evaluation can improve summary quality without requiring costly fine-tuning or retrieval-augmented architectures, making the approach more feasible for resource-limited deployment environments. This prompt-level feedback mechanism is conceptually analogous to reinforcement learning from human feedback but operates entirely through clinician-interpretable error categories, thereby preserving transparency and auditability. The convergence behavior observed across iterations suggests that a stable minimum-error configuration can be achieved within a clinically feasible number of refinement cycles. In practice, refinement was stopped after 15 iterations because subsequent cycles produced only marginal qualitative improvement relative to the operational cost of repeated generation and clinician review.

The MORPHEUS pipeline generated complete discharge summaries at a mean of 62.7 s per patient (Stage 1). In the current implementation, orchestration, preprocessing, scoring, and workflow management were performed locally on edge hardware, while language generation inference was performed through the OpenAI API. Based on input from clinical staff at the participating NICU, manual discharge summary preparation typically requires approximately 10–40 min per patient, depending on case complexity and length of stay, even within the current EMR-based workflow. This observation is consistent with broader evidence that discharge documentation is time-intensive and that delayed completion of discharge summaries remains common in hospital practice [44]. The observed reduction in documentation time could translate into substantial recovery of clinician-hours annually in high-volume NICUs, with potential implications for clinician workload and burnout. The automated evaluation stage adds approximately 60–90 s per patient and runs asynchronously, functioning as a quality assurance mechanism rather than a workflow bottleneck. Accordingly, the term ‘edge-hosted’ in this study refers primarily to local orchestration, PHI-controlled workflow execution, and hospital-side data handling rather than fully local LLM inference.

Deployment on the NVIDIA Jetson AGX Orin reflects a deliberate design choice for settings where local orchestration, data control, and modest infrastructure requirements are important (Supplementary Table S2). In the current implementation, orchestration and workflow execution were performed on edge hardware, while runtime was still influenced substantially by network delay and waiting time associated with OpenAI API calls. The decision to use a fixed OpenAI model in this study reflected a pragmatic focus on prompt architecture and documentation quality before benchmarking fully local inference. Future migration to locally hosted quantized models may reduce latency and eliminate per-call API cost, but this will require direct benchmarking in the target hardware environment. In the current implementation, generation required approximately 18,000–25,000 tokens per patient, corresponding to an estimated cost of USD $0.03–$0.07 per discharge using cloud LLM APIs; including automated evaluation, the total per-patient cost was estimated at USD $0.08–$0.17.

The field-level rubric developed here (72 candidate clinical fields across section contexts, scored across five orthogonal dimensions) represents a contribution independent of the generation system. Existing approaches to LLM documentation evaluation rely predominantly on holistic or aggregate scoring, which can mask deficits in completeness or safety. The field-dimension matrix used here reduces this masking effect and produces actionable, section-specific error signals. The rubric is likely adaptable to other NICU documentation contexts and, with modification, to other high-acuity inpatient settings where discharge summary quality has direct implications for continuity of care [45].

Several limitations merit consideration. First, the 8000-character input truncation applied in Stage 1 preferentially retains the most recent source text, which may omit clinically significant early events in long admissions. This tradeoff favored computational feasibility and reduced API transaction cost but may have reduced recovery of clinically important early-admission events in prolonged NICU hospitalizations. This likely contributed to residual omission errors, particularly in vitals, medications, procedures, jaundice-related content, nursing notes, and investigation summaries. Second, Stage 1.5 applied LLM-based backfill to nearly all absent sections without distinguishing missing documentation from conditionally irrelevant sections. For domains such as ROP screening, CNS complications, and death notes, absence often reflects an appropriate clinical context. Future iterations should incorporate clinical relevance gating and confidence-aware review mechanisms before triggering refinement. Third, although orchestration and PHI-controlled workflow execution occurred locally on edge hardware, language generation inference in the current implementation depended on cloud-based API access. Future evaluation using fully local hospital-deployable LLMs will be important to assess latency, operational cost, and deployment feasibility in restricted-network environments. Fourth, this study was conducted at a single Level III NICU in India, and generalizability to other settings with different EMR platforms, documentation cultures, or patient populations requires prospective evaluation [46,47]. Because the MORPHEUS pipeline depends partly on institution-specific documentation structure, section organization, and longitudinal note patterns, prospective validation across heterogeneous NICU environments and EMR systems will be necessary before broader deployment claims can be made. Finally, clinician involvement was limited to rubric design and validation; the system has not yet been evaluated in a prospective workflow study with real-time clinician interaction, which remains an essential next step before clinical adoption.

Future work will extend the platform to incorporate bedside video and multimodal physiologic data captured through the NEO module, enabling automated procedure documentation, behavioral assessment, and workflow interpretation from direct observation. Future versions of the pipeline may also incorporate audience-specific summary generation, where discharge summaries are adapted according to the educational level, language preference, and domain understanding of the target reader. Parent-facing summaries using simplified and lay-language phrasing while remaining faithful to the clinical record represent a near-term extension with direct relevance to family-centered NICU care. Additional planned directions include deployment and benchmarking of fully local hospital-deployable LLMs on edge hardware, implementation of clinical relevance gating and confidence-aware review mechanisms, and expansion of the framework beyond discharge summaries into other NICU documentation workflows such as daily progress notes, nutrition summaries, respiratory-support documentation, procedure summaries, and longitudinal developmental follow-up reporting.

## 5. Conclusions

In this single-center NICU study, MORPHEUS-generated discharge summaries achieved higher structured rubric-based scores and lower omission burden than clinician-authored summaries across all five evaluated dimensions—accuracy, completeness, coherence, actionability, and non-hallucination. Iterative prompt refinement significantly reduced omission burden without model fine-tuning, while other gains across iterations were supportive but not independently significant. The field-level rubric provides an auditable framework for evaluating AI-generated clinical documentation and identifying specific residual failure modes. Prospective workflow validation and evaluation of locally hosted inference are the next steps toward responsible deployment in neonatal care.

## Figures and Tables

**Table 1:** Comparative synthesis of prior LLM-assisted discharge documentation studies and related clinical documentation tasks.

Study	Primary Task	Setting	Model/ Pipeline	Study Approach	Main Findings and Relevance to Present Study
Woo et al., 2025/2026 [13]	Adjacent task: scoping review	Cross-setting	Review of 41 studies	Mapped LLM applications in clinical documentation; many studies focused on note generation, discharge summaries, and encounter documentation; common issues included hallucinations, complex-case performance, privacy, and clinician trust	Provided broader contextual support for LLM-assisted clinical documentation research by summarizing reported benefits in efficiency, readability, and standardization across multiple healthcare settings. The review also highlighted persistent concerns regarding hallucinations, factual reliability, clinician trust, privacy, and degraded performance in clinically complex cases, reinforcing the importance of grounded evaluation and safety-focused summarization workflows.
Butt et al., 2024 [14]	Adjacent task: NICU documentation standardization	NICU	Standardized progress-note template and feedback system	Improved NICU documentation structure/compliance through template redesign and feedback	Reinforced that neonatal documentation is structurally unique and benefits from standardized organization and feedback-driven workflows. However, the work focused on template compliance and documentation consistency rather than automated synthesis of prolonged neonatal hospital courses across heterogeneous EMR sources.
Williams et al., 2025 [15]	Discharge summary generation	Adult general inpatient; UCSF hospital discharge summaries	LLM-generated discharge narratives compared with physician-authored narratives	Blinded comparative quality and safety study of 100 discharge summaries; LLM and physician summaries were rated comparably overall, with LLM outputs more concise/coherent but less comprehensive	Demonstrated feasibility of blinded clinician evaluation of LLM-generated discharge summaries in real clinical workflows, with strengths in coherence and conciseness. However, residual gaps in comprehensiveness emphasized the challenge of preserving clinically important details during automated summarization.
Lyu et al. (UF-HOBI), 2024 [16]	Discharge summary generation	Shared-task benchmark, not NICU-specific	Two-stage hybrid pipeline: NER extraction + prompt-tuned GatorTronGPT	Generated two discharge sections (“Brief Hospital Course” and “Discharge Instructions”); ranked 5th with overall score 0.284	Relevant for demonstrating hybrid structured-generation approaches and handling dispersed records. However, the study evaluated selected discharge components rather than complete discharge synthesis and did not assess clinician-grounded safety, longitudinal neonatal complexity, or field-level documentation completeness.
Klang et al., 2025 [17]	Discharge summary generation	Adult inpatient discharge summaries	Prompt-engineering strategy: Summarize-then-Prompt	Evaluated whether summarizing individual notes before final prompting improves discharge summary generation	Supports the importance of prompt architecture and iterative summarization strategies for long clinical records. However, the work did not address the role of prompt orchestration and staged synthesis approaches for handling temporally distributed documentation.
Mehri et al., 2026 [20]	Automated discharge summary generation and evaluation	Dutch academic hospital; multi-specialty inpatient setting	EHR-integrated GPT-4o discharge summary generation pipeline	Compared physician-written and AI-generated discharge summaries across multiple specialties using blinded clinician evaluation	Demonstrated that EHR-integrated LLM-generated discharge summaries can achieve quality comparable to physician-written summaries in real-world workflows, although completeness gaps and need for specialty-specific refinement remained. Supports the importance of clinically grounded evaluation frameworks and structured prompting approaches.
Rust et al., 2025 [21]	Adjacent task: simplification/patient-facing adaptation	Adult cardiology discharge summaries	GPT-4o, full-text vs. segment-wise prompting	Simplified existing discharge summaries and generated lifestyle recommendations; improved readability and was rated largely correct/complete/harmless by experts	Demonstrates value of section-wise prompting and discharge-document adaptation workflows. Supports the importance of modular prompting strategies for transforming complex clinical narratives into targeted outputs for different audiences.
Mudumbai et al., 2025 [18]	ICU discharge summary generation/evaluation	ICU setting	LLM evaluation for clinically relevant ICU discharge summaries	Evaluated LLM performance on ICU discharge summary generation, emphasizing the challenge of summarizing complex ICU courses	Particularly relevant because ICU workflows are closer to NICU complexity than general inpatient settings. However, adult ICU workflows differ substantially from neonatal care, and available reports did not establish NICU-specific templates, longitudinal neonatal constraints, or clinician-grounded field-level evaluation.
Hains et al., 2025 [19]	Discharge summary preparation/generation	Real-world EMR data; adult hospital setting	LLM-based discharge summary preparation from EMR data	Demonstrated promise using real-world EMR-derived records for discharge summary preparation	Operationally relevant because it used authentic EMR-derived discharge workflows. However, published evaluation details on section-level safety, omission analysis, and longitudinal complexity remained limited.

**Table 2:** Fields for neonatal discharge summary evaluation rubric.

Initial Assessment			
Presenting problem and relevant antenatal/perinatal context, Delivery details and APGAR scores if available, Immediate postnatal events, Admission anthropometry (weight, length, OFC), Initial vitals (HR, RR, SpO_2_, BP, CRT), Early systemic examination findings and initial differential impression.			
Feeding and Nutrition	Medications	Infections	CNS
Feeding initiation Feeding mode PN/EN timeline Advancement pattern Max tolerated feed Growth Velocity (g/kg/day)	Drug name Route Dose correctness Duration	Suspected/confirmed Sepsis workup Antibiotics timeline Culture result Duration of therapy Source of suspected infection (maternal, device, community, hospital)	Tone and reflex exam Seizure documentation Neuroimaging Neurological impression Tone evolution direction (improving/stable/worsening)
Vitals	Respiratory Distress Summary	Jaundice	Apnea
HR range RR range SpO_2_ BP Temperature CRT	Onset timing Respiratory mode(s) used FiO_2_ range Escalation/weaning timeline Surfactant use Respiratory severity scale (Silverman/Downes)	Onset timing Phototherapy type Phototherapy timeline Bilirubin values Threshold interpretation Exchange transfusion status	Onset timing Frequency/severity Intervention documented Resolution timing Trigger identifier (central/obstructive/secondary cause)
Shock	ROP	Procedures	Investigations
Hypoperfusion signs Shock intervention Resolution timing Type (hypovolemic/septic/ cardiogenic)	Screening timing Zone Stage Plus disease Intervention and follow-up PMA (Post-Menstrual Age) at screening	Procedure type Indication Date/timeline Outcome/status	Hematology Metabolic Microbiology Imaging Abnormal values

Highlighted background colors are used to visually group related neonatal discharge summary domains and rubric sections for easier interpretation and organization of evaluation fields.

**Table 3:** Scoring Framework for 5 dimensions.

Clinical Accuracy	Completeness/Missing Data Detection	Actionability and Continuity of Care
1.0 = Factually correct, clinically plausible, matches chart, values. 0.5 = Minor vagueness, non-specific phrasing, rounding or omission of units, but clinically acceptable. 0 = Factually incorrect, physiologically impossible, contradicts chart/evidence, or misinterprets findings.	1.0 = All required sub-elements documented with meaningful detail and measurable values when applicable. 0.5 = ≥50% elements present OR partially descriptive without quantification. 0.0 = Largely missing placeholders, generic descriptors, or value-less text.	1.0 = Provides a clear next step, monitoring instruction, follow-up requirement, threshold or escalation trigger. 0.5 = Gives some guidance but lacks specificity, interval, or responsible provider/location. 0.0 = Descriptive without implied clinical decision or continuity relevance.
Coherence and Timeline Validation	Non-Hallucination Scoring	
1.0 = Chronicle flows logically, dates/DOL consistent across the entire summary, no reverse-time events, and resolution follows intervention. 0.5 = Timeline implied but not explicit OR mild ambiguity without contradiction. 0.0 = Contradictory sequences, impossible chronology, or circular logic.	1.0 = Fully traceable to primary record, timestamp, nursing/doctor notes, orders, reports, or validated clinical logic. 0.5 = Ambiguous origin OR seems inferred rather than documented, but possibly true. 0.0 = Invented, guessed, reverse-engineered, or clearly absent from the chart.	

Highlighted background colors are used to visually distinguish and group related scoring dimensions in the evaluation rubric.

**Table 4:** Gestation-wise Baseline Characteristics (N = 401).

Characteristics	Gestational Age Groups, Weeks				
	23–25 (n = 3)	26–28 (n = 12)	29–31 (n = 40)	32–34 (n = 65)	≥35 (n = 281)
Gestation *	23.9 (0.0)	26.2 (0.9)	29.9 (0.8)	32.9 (0.9)	37.2 (1.6)
Birth weight (grams) *	630.0 (26.5)	820.4 (126.8)	1138.7(306.8)	1749.0 (426.2)	2691.9 (555.7)
Gender (Male)	2 (66.7%)	9 (75.0%)	30 (75.0%)	41 (63.1%)	175 (62.3%)
Conception type (IVF)	2 (66.7%)	1 (8.3%)	5 (12.5%)	15 (23%)	21 (7.4%)
Length of Stay (LOS) #	17.7 (15.5)	59.5 (63.5)	35.3 (50.75)	14.2 (15)	7.7 (9)
Antenatal Steroids	0 (0.0%)	8 (66.7%)	27 (67.5%)	38(58.4%)	36(12.8%)
Mode of delivery (LSCS)	2 (66.7%)	7 (58.3%)	32 (80.0%)	55 (84.6%)	214(76.5%)
Outborn	2 (66.7%)	2 (16.7%)	17 (42.5%)	17 (26.15%)	82 (29.18%)
Baby type (Multiple)	2(66.7)	2(16.7%)	7 (17.5%)	16 (24.6%)	26 (9.25%)
APGAR #—One minute	6.0(0.0)	5.2 (1.5)	5.8 (1.0)	7.4 (1.0)	7.7 (2.0)
APGAR #—Five minutes	7.0(0.0)	7.2 (1.75)	7.8 (2.0)	8.8 (2.0)	9.0 (1.0)
Birth head circumference	21.5(1.1)	23.9 (1.1)	27.5 (2.5)	29.9 (2.9)	33.1 (2.1)
Jaundice needing phototherapy	NA	2 (16.7%)	1 (2.5%)	11 (16.9%)	104 (37.3%)
Sepsis	3 (100.0%)	4 (30.8%)	11 (30.6%)	17 (27.4%)	50 (17.9%)
Respiratory Distress Syndrome	3 (100.0%)	11 (84.6%)	35 (97.2%)	52 (83.9%)	154 (55.2%)

Data expressed as n (%) unless specified * Mean (Standard Deviation), # median (IQR), LOS: Length of Stay, IVF: In Vitro Fertilization, LSCS: Lower segment Caesarean section, APGAR: Appearance, Pulse, Grimace, Activity, and Respiration.

**Table 5:** Stage-wise results.

	Stage 1			Stage 1.5		
Time per patient (seconds)	62.73			169.5		
LLM Calls (per patient/total in stage)	12/4950			7/2642		
Sections	Patient with these sections	Mean time (seconds)	Mean number of characters	Number of sections refined	Average time per section (seconds)	Mean number of characters
Vitals	401	2.61	22,117	19	43.31	71,570
Medications	401	7.06	16,688	251	4.36	75,196
Nursing notes	401	NA	43,161	0	NA	NA
Feeding and Nutrition	401	5.02	11,651	0	NA	NA
Procedures	277	5.61	3670	2	21.1	79,721
Initial Assessment (birth, antenatal, status at admission)	400	1.57/ 2.44/ 5.79	1273	0	NA	NA
Demographics	401	10.07	543	0	NA	NA
Investigation summary	372	18.63	220,171	26	0	14
Respiratory distress summary	100	1.78	21,994	121	116.21	57,757
Assessments (CNS, shock, apnea, infection, jaundice, ROP)	0, 34, 6, 78, 190, 0	0, 3.5, 1.5, 3.6, 1.21, 0	52,858, 53,060, 52,873, 52,858, 54,791, 0	106, 148, 203, 144, 28, 17	39.88, 44.07, 34.55, 12.31, 24.25, 30.79	90,892, 86,952, 81,050, 101,697, 70,291

**Table 6:** Results with respect to data dimensions in each iteration.

Dimension	Total Fields Compared ^	Human	LLM	Reward/Penalty/Neutral
Clinical Accuracy	3142	0.75	**0.95**	842/141/2159
Completeness	3129	0.67	**0.92**	1157/177/1795
Actionability	2614	0.72	**0.94**	801/122/1691
Coherence	2616	0.74	**0.95**	721/105/1790
Non-hallucination	2619	0.77	**0.96**	610/79/1930

* (per patient 72 fields for each dimension * 401 patients theoretical maximum = 28,872 field slots per dimension). ^ field-level paired comparisons that were scorable for that dimension.

**Table 7:** Error Taxonomy comparison between LLM and human-generated discharge summaries.

Error Type	LLM Total	Human Total	LLM Rate/ Patient	Human Rate /Patient	LLM Rate /100 Fields	Human Rate /100 Fields	Relative Reduction (%)	*p*-Value
Omission	**1010**	2703	**2.519**	6.74	**3.498**	9.362	**62.63**	**<0.001**
Unsupported assertion	**31**	54	**0.077**	0.135	**0.107**	0.187	**42.59**	**0.017**
Contradiction	**43**	112	**0.107**	0.279	**0.149**	0.388	**61.61**	**<0.001**

Bold formatting highlights statistically significant *p*-values (*p* < 0.05) and corresponding section-level findings for ease of interpretation.

**Table 8:** Top five sections contributing the highest combined error burden in MORPHEUS-generated and clinician-authored discharge summaries.

	Omission		Unsupported		Contradiction		Total Errors	
Section	LLM	Human	LLM	Human	LLM	Human	LLM	Human
Vitals	119	1032	3	9	26	32	148	1073
Medications	409	489	3	19	10	34	422	542
Investigation Summary	332	350	9	12	1	10	342	372
Procedures	20	342	3	4	2	13	25	359
Jaundice	32	107	2	2	2	2	36	111

**Table 9:** Section-level performance comparison between final AI-generated and clinician-authored summaries.

Section	AI Mean ± SD	Clinician Mean ± SD	Mean Difference	95% CI	*p*-Value	Relative Improvement (%)
Apnea	**0.007 ± 0.086**	0.057 ± 0.307	0.05	0.019 to 0.080	**0.002**	86.96
CNS	**0.045 ± 0.230**	0.145 ± 0.359	0.1	0.061 to 0.138	**<0.001**	68.97
Feeding And Nutrition	**0.007 ± 0.086**	0.157 ± 0.503	0.15	0.101 to 0.198	**<0.001**	95.24
Infections	**0.032 ± 0.191**	0.065 ± 0.293	0.032	0.002 to 0.063	**0.036**	50.0
Initial Assessment	**0.022 ± 0.179**	0.162 ± 0.460	0.14	0.092 to 0.187	**<0.001**	86.15
Investigation Summary	**0.853 ± 1.103**	0.928 ± 1.193	0.075	−0.059 to 0.208	0.417	8.06
Jaundice	**0.090 ± 0.286**	0.277 ± 0.534	0.187	0.130 to 0.244	**<0.001**	67.57
Medications	**1.052 ± 1.338**	1.352 ± 1.341	0.299	0.147 to 0.452	**<0.001**	22.14
Procedures	**0.062 ± 0.330**	0.895 ± 1.403	0.833	0.697 to 0.969	**<0.001**	93.04
ROP	**0.082 ± 0.275**	0.100 ± 0.308	0.017	−0.017 to 0.052	0.317	17.5
Respiratory Distress Summary	**0.017 ± 0.131**	0.192 ± 0.530	0.175	0.125 to 0.224	**<0.001**	90.91
Shock	**0.062 ± 0.242**	0.150 ± 0.378	0.087	0.046 to 0.129	**<0.001**	58.33
Vitals	**0.369 ± 0.666**	2.676 ± 2.229	2.307	2.094 to 2.520	**<0.001**	86.21

Bold formatting highlights statistically significant *p*-values (*p* < 0.05) and corresponding section-level findings for ease of interpretation.

**Table 10:** Impact of iterative prompt refinement on performance and safety metrics (temperature = 0, top_p = 1, seed = 42, time out = 270 s, number of iterations = 15).

Metric	Iteration 1	Iteration 15	Absolute Change	% Change	*p*-Value
Accuracy	0.937	0.943	0.006	0.6	0.124
Completeness	0.913	0.933	0.020	2.0	0.262
Coherence	0.941	0.961	0.020	2.0	0.520
Actionability	0.935	0.957	0.022	2.2	0.892
Non-hallucination	0.952	0.974	0.022	2.2	0.670
Omission rate/patient	2.484	1.807	−0.677	−27	0.028
Unsupported assertions/patient	0.095	0.072	−0.023	−24	0.317
Contradictions/patient	0.095	0.095	0	0	0.8842

**Table 11:** Final AI-versus-clinician comparison at the dimension level.

Dimension	LLM Mean (SD)	Human Mean (SD)	Mean Difference	95% CI	*p*-Value	Cohen’s d
Accuracy	**0.927 (0.106)**	0.748 (0.231)	0.178	0.154–0.201	<0.01	**0.742**
Completeness	**0.911 (0.124)**	0.671 (0.230)	0.24	0.202–0.251	<0.01	**0.90**
Coherence	**0.935 (0.110)**	0.741 (0.264)	0.19	0.163–0.217	<0.01	**0.7**
Actionability	**0.931 (0.134)**	0.724 (0.265)	0.202	0.174–0.229	<0.01	**0.734**
Non-hallucination	**0.951 (0.137)**	0.784 (0.259)	0.161	0.135–0.188	<0.01	**0.61**

Bold formatting highlights statistically significant effect sizes (Cohen’s d) and corresponding dimension-level findings for ease of interpretation.

**Table 12:** Pipeline-Stage Ablation Analysis of the MORPHEUS NICU Discharge Summary Generation Framework Across Progressive Prompting and Refinement Configurations.

Metric	Human Summaries	Stage 0: Generic Single-Stage Prompt	Stage 1: Section-Wise Generation	Stage 1.5: Targeted Refinement	Stage 2: Final MORPHEUS Summary
Accuracy	0.748	0.858	0.953	0.961	**0.956**
Completeness	0.671	0.794	0.935	0.959	**0.939**
Coherence	0.741	0.842	0.965	0.961	**0.966**
Actionability	0.724	0.840	0.962	0.959	**0.962**
Non-hallucination	0.784	0.875	0.970	0.988	**0.974**
Omissions/patient	7.64	5.12	3.00	2.76	**3.16**
Unsupported/patient	0.16	0.00	0.12	0.00	**0.08**
Contradictions/patient	0.36	0.16	0.36	0.08	**0.08**

Bold formatting indicates the final Stage 2 MORPHEUS discharge-summary configuration evaluated in the primary manuscript analysis.

## Data Availability

The raw data used in this study consists of protected neonatal patient health information (PHI), including longitudinal NICU clinical records and discharge documentation governed by hospital privacy policies, institutional approvals, and ethical restrictions. Due to patient confidentiality obligations and institutional data-sharing limitations, the raw clinical data cannot be publicly shared.

## Supplementary Materials

The following supporting information can be downloaded at: https://www.mdpi.com/article/10.3390/healthcare14111457/s1.

## Abbreviations

The following abbreviations are used in this manuscript:

AI	Artificial Intelligence
ARCHITECT	Bedside data acquisition system name used in the study
BP	Blood Pressure
CC BY	Creative Commons Attribution
CI	Confidence Interval
CNS	Central Nervous System
DOL	Day of Life
EMR	Electronic Medical Record
FiO_2_	Fraction of Inspired Oxygen
ICU	Intensive Care Unit
HR	Heart Rate
I/O	Intake and Output
IoT	Internet of Things
IQR	Interquartile Range
LLM	Large Language Model
MORPHEUS	Automated discharge summary generation pipeline name used in the study
MRN	Medical Registration Number
NEO	Embedded audio–video module name used in the platform
NICU	Neonatal Intensive Care Unit
NVMe	Non-Volatile Memory Express
pCO_2_	Partial Pressure of Carbon Dioxide
pH	Potential of Hydrogen
ROP	Retinopathy of Prematurity
RR	Respiratory Rate
SD	Standard Deviation
SpO_2_	Peripheral Capillary Oxygen Saturation
SSD	Solid-State Drive
TB	Terabyte
