# Supplementary material for: Edge-Hosted LLM-Assisted NICU Discharge Summary Generation: Field-Level Evaluation Using a Clinician-Defined Rubric
Source: Healthcare (Basel). 2026 May 25;14(11):1457. doi: 10.3390/healthcare14111457 (PMC13257403; doi:10.3390/healthcare14111457)
Supplement: Supplementary file 1 [file healthcare-14-01457-s001.zip › healthcare-4269874-supplementary.pdf]

Supplementary Information

FILE S1

Discharge Summary

Name: Sex: UHID No.:
DOB: TOB:
DOA: DOD:
Address:

Anthropometry
Birth Weight:____gms
Adm Weight: gms Disch Wt: gms
OFC at adm: cms OFC at Disch: cms
Length at adm: cms Length at Disch: cms

Final Diagnosis:

Advice on Discharge:

To attend Nursery/ Pediatric Emergency on noticing warning signs like cyanosis/ difficulty in breathing/ refusal of feeds/ abnormal movements. Please contact NICU (Nursery) Helpline No. XXXXXXXXXX or XXXXXXXXX

Follow up:

Initial Assessment

Course during Hospital:
1. Vitals
2. Respiratory Distress Summary
3. Jaundice
4. Apnea
5. Infections
6. CNS
7. Shock
8. ROP
9. Feeding and Nutrition
10. Medications
11. Procedures
12. Investigation summary

Resident: Consultant

Supplementary Table S1: Clinically meaningful discrepancies identified in discharge summaries.

Category	Example Change	Example Description
Omission – Vitals	Add explicit BP, perfusion, and CRT values rather than generic narrative text.	Vital-sign details were frequently omitted despite clear chart evidence. Typical examples included missing blood pressure values and capillary refill time even when the chart documented them explicitly.
Omission – Medications	Include structured medication details: name, dose, route, frequency, and duration.	Medication discrepancies commonly reflected missing route, dose, or duration. Typical examples included omission of IV route or exact milligram dose despite explicit medication entries in the source chart.
Unsupported assertion – Investigation Summary	Restrict laboratory interpretation to chart-supported hematology, metabolic, microbiology, and imaging findings.	Investigation Summary remained challenging for both summary types. Errors included unsupported laboratory interpretations or incomplete reporting of hematology, bilirubin, glucose, electrolyte, or microbiology results documented in the chart.
Contradiction – Procedures / Status	Ensure procedural status and outcome statements exactly match the chart; avoid inferential narrative.	Procedural contradictions arose when summary text conflicted with charted status. Typical examples included mismatch in ventilatory support status or procedural outcome, where the chart and summary described different clinical states.
Omission – Jaundice	Include bilirubin values and phototherapy timeline whenever present in the chart.	Jaundice-related errors commonly reflected missing bilirubin measurements or phototherapy details. Summaries often failed to carry forward explicit total/direct bilirubin values or treatment timing recorded in the source notes.

Supplementary Table S2: Local versus cloud responsibilities in MORPHEUS pipeline

Component	Executed Locally (Jetson)	Executed via OpenAI API
EMR ingestion	Yes	No
Prompt assembly	Yes	No
Workflow orchestration	Yes	No
Retry handling	Yes	No
Rubric scoring pipeline	Yes	No
LLM text generation	No	Yes
Final assembly	Yes	No

Supplementary Table S3: Clinician manual review findings across representative cases

Case	Major Clinical Domain Reviewed	Omissions Identified	Hallucination / Unsupported Inference	Temporal Consistency Issues	Medication / Procedure Errors
1	Sepsis / Feeding	Missing PN details before EN initiation	Tobramycin likely inferred incorrectly as systemic antibiotic instead of eye drops	Mild DOL inconsistencies	Medication indication unclear
2	Respiratory / Feeding	Missing direct feeding transition	None	None	None

Case	Major Clinical Domain Reviewed	Omissions Identified	Hallucination / Unsupported Inference	Temporal Consistency Issues	Medication / Procedure Errors
3	Feeding / Medications	Missing early feeding course before DOL 2	None	Mild sequencing issues	Medication spelling corrections
4	Infection / Procedures	Missing final sepsis status	Possible over-interpretation of clinical sepsis severity	Some unclear progression	Procedure duplication
5	Prematurity / Apnea / Sepsis	Missing structured sepsis summary	Some unsupported escalation narratives	Multiple DOL inconsistencies	Medication summarization needed
6	ROP / CNS / Shock	Missing neurologic and shock synthesis	Repeated hemodynamic interpretations	None	Medication placement issues
